# Breeding behavior of female white‐tailed deer relative to conception: Evidence for female mate choice

**DOI:** 10.1002/ece3.2845

**Published:** 2017-03-12

**Authors:** Jeffery D. Sullivan, Stephen S. Ditchkoff, Bret A. Collier, Charles R. Ruth, Joshua B. Raglin

**Affiliations:** ^1^ School of Forestry and Wildlife SciencesAuburn UniversityAuburnALUSA; ^2^ School of Renewable Natural ResourcesLouisiana State University Agricultural CenterBaton RougeLAUSA; ^3^ South Carolina Department of Natural ResourcesColumbiaSCUSA; ^4^ Norfolk Southern RailwayDorchesterSCUSA

**Keywords:** activity pattern, breeding strategy, excursions, mate choice, white‐tailed deer

## Abstract

Female white‐tailed deer (*Odocoileus virginianus*) are thought to choose between two behavioral strategies to maximize the quality of potential mates: sit and wait, characterized by concentrating activity within a restricted area, and excursive behavior, characterized by increased activity and excursions outside the home range. As movement patterns may influence conception, our goal was to examine the patterns of female white‐tailed deer movements to evaluate which breeding strategy was employed. We equipped 36 female white‐tailed deer with GPS collars from August 2013 to December 2015. We found that movement rate and probability of activity were greatest near the peak of the breeding season, and we observed increases in both metrics during the 40 days prior to estimated conception. Peak size of home range and core area occurred in the days surrounding conception. We found that 11 deer performed an excursion, ranging from 43 days before until 36 days after conception, with the peak probability of being outside of an individual home range occurring 1 day prior to conception. Our results suggest that female white‐tailed deer may attempt to maximize the quality of their mates by advertising availability for breeding through excursive behaviors just prior to entering estrus.

## 1 Introduction

An animal's fitness, or the extent to which it contributes genetic material to subsequent generations, is determined by reproductive success and the success of progeny (Freeman & Herron, 2004). Due to inequalities in parental investment found in polygynous species (Maynard Smith, 1977; Raven & Johnson, 2001; Trivers, 1972), advantageous methods to increase fitness may vary between the sexes. Male reproductive output is limited only by breeding success, and as sperm is relatively inexpensive to produce, males can increase fitness by minimizing selectivity and breeding as many females as possible (Raven & Johnson, 2001). Females face greater investment due to the demands of gestation and lactation (Clutton‐Brock, 1989; Speakman, 2008) and possible trade‐offs between current and future reproduction (Del Guidice, Gangestad, & Kaplan, 2015; Stearns, 1992). Therefore, a female can improve fitness by maximizing mate quality, generally achieved through a high degree of selectivity (Raven & Johnson, 2001). By breeding with a high‐quality male, females provide offspring with advantages in areas such as future reproductive potential and immunocompetence (Ditchkoff, Lochmiller, Masters, Hoofer, & Van Den Bussche, 2001; von Schantz, Grahn, & Goransson, 1994). However, as females near the end of receptivity, fitness benefits accrued from breeding with a high‐quality male may be lessened relative to benefits of conceiving during first estrus (predator swamping and increased growth of offspring before their first winter; Zwank & Zeno, 1986; Whittaker & Lindzey, 1999; Gray, Ditchkoff, Causey, & Cook, 2002), leading her to breed with a lower‐quality male rather than risk not reproducing in the first estrous cycle.

Due to the benefits of breeding with a high‐quality male, behavior of females approaching receptivity is likely a function of herd demography. In white‐tailed deer (*Odocoileus virginianus*), previous studies have documented two behavioral strategies employed by females during the breeding season. The first strategy, known as “sit and wait,” has been observed near the peak of the breeding season and is characterized by a decrease in movement rate and range size, and an increase in core area use (Beier & McCullough, 1990; Holzenbein & Schwede, 1989; Ivey & Causey, 1981). The sit‐and‐wait strategy is advantageous for the female as it provides reduced energy expenditure and limits mortality risk by remaining in a familiar area (Labisky & Fritzen, 1998). Sit‐and‐wait behavior theoretically allows a female to be located by roaming males, as she is in a predictable location well marked with indicators expressing receptivity (Beier & McCullough, 1990; Holzenbein & Schwede, 1989; Labisky & Fritzen, 1998). Therefore, the sit‐and‐wait strategy is theorized to most likely occur in populations with high deer densities and high male:female ratios (Beier & McCullough, 1990; Holzenbein & Schwede, 1989; Labisky & Fritzen, 1998).

A second behavioral strategy used by female white‐tailed deer during the breeding season is excursive behavior. Excursive behavior is generally characterized by increased activity, leading to an excursion outside of the home range (D'Angelo et al., 2004; Kolodzinski et al., 2010; Sawyer, 1981). Previous work has described these excursions as brief, lasting an average of 24 hr, and variable in distance, ranging from 0.57 to 4.78 km (Kolodzinski et al., 2010). Excursive behavior may improve a female's opportunity of encountering a mate during receptivity (Ozoga & Verme, 1975), particularly in populations that are characterized by low density or a female‐biased sex ratio (Beier & McCullough, 1990; D'Angelo et al., 2004; Holzenbein & Schwede, 1989; Kolodzinski et al., 2010; Labisky & Fritzen, 1998). Excursive behavior has also been documented in other cervids such as roe deer (*Capreolus capreolus*; Lovari, Bartolommei, Meschi, & Pezzo, 2008; Richard et al., 2008; Debeffe et al., 2014) and red deer (*Cervus elaphus*; Stopher et al., 2011). Excursive behavior may be an effort to maximize the probability of finding a male during the short period that the female is receptive (D'Angelo et al., 2004; Holzenbein & Schwede, 1989; Labisky & Fritzen, 1998). However, excursions have been documented in populations with both high (Ivey & Causey, 1981; Kolodzinski et al., 2010) and low densities (Labisky & Fritzen, 1998; D'Angelo 2004), leading some to conjecture that excursive behaviors during the breeding season may be a form of direct female mate choice (Hasapes, 2012; Kolodzinski et al., 2010), or a means to incite male competition and access a higher‐quality mate (Cox & Le Boeuf, 1977).

Unfortunately, there still exists a major gap in our understanding of deer breeding behavior. Each study that has previously documented the movement patterns of free‐ranging female white‐tailed deer during the breeding season has performed so only at the broad scale of population breeding stages (i.e., prerut, rut, postrut; Sawyer, 1981; Holzenbein & Schwede, 1989; Beier & McCullough, 1990; Labisky & Fritzen, 1998; D'Angelo et al., 2004; Kolodzinski et al., 2010). While the aforementioned works have identified general behavioral patterns of females during the breeding season, the behavioral and spatial tendencies of females relative to their specific conception date remain unknown. Therefore, evaluating the movement of female white‐tailed deer relative to conception is a clear next step in understanding not only how female large mammals utilize space during a key life‐history event, but also in exploring the possibility of female mate choice. The goal of this project was to examine the movement and space use patterns of female white‐tailed deer during periods relevant to breeding at the population and individual levels. Our objectives were to: (1) characterize activity patterns of free‐ranging female white‐tailed deer relative to the populations’ peak of conception, (2) characterize activity patterns relative to individual conception, and (3) determine whether the observed behaviors are breeding related.

## 2 Materials and Methods

### 2.1 Study area

Our research was conducted at Brosnan Forest, a 5,830‐ha tract of lower coastal plain habitat in Dorchester County, South Carolina (N 33′08.951, W 80′25.726), and took place exclusively on the 2,552‐ha portion of the property located north of Highway 78. Deer density on this property had been estimated at 20 per km^2^ with a male to female ratio of 1:1.4 (J.B. Raglin, Norfolk Southern Railway, personal commun., April 2016). McCoy, Ditchkoff, Raglin, Collier, and Ruth (2013) reported a peak of conception on 9 October for Brosnan Forest, with 80% of conceptions occurring between 19 September and 28 October. Approximately 93% forested, the study area contained mostly open longleaf pine (*Pinus palustris*) stands interspersed with mixed hardwoods (Collier, Ditchkoff, Raglin, & Smith, 2007). Hardwood drains were found throughout the property with mixed pine‐hardwood areas comprised of loblolly (*Pinus taeda*), slash (*Pinus elliottii*), and pond (*Pinus serotina*) pine, along with oak (*Quercus* spp.), sweetgum (*Liquidambar styraciflua*), and red maple (*Acer rubrum*). Bottomland drains included oak, sweetgum, black gum (*Nyssa sylvatica*), and yellow poplar (*Liriodendron tulipifera*). The majority of forest stands were actively managed for wildlife and timber production and were burned on a 2‐ to 3‐year rotation to maintain an open understory (Collier et al., 2007; Lauerman, 2007). Food plots on the study area ranged in size from 0.6 to 8.1 ha and comprised a total of 119 ha. While a majority of plots were planted annually with a cool season mix of various clovers (*Trifolium* spp.), grains (oats, *Avena fatua*; wheat, *Triticum aestivum*), chicory (*Cichorium intybus*), and winter peas (*Pisum sativum*), additional plots received spring plantings of soybeans (*Glycine max*), sorghum (*Sorghum bicolor*), or game bird mix containing sorghum (*Sorghum bicolor*), buckwheat (*Fagopyrum esculentum*), benne (*Sesamum indicum*), and sunflower (*Helianthus* spp.). There were also ~55 feeders distributed throughout the study area dispensing shelled corn during the hunting season which, although it ran from 15 August to 1 January in this portion of South Carolina, did not begin until 15 September on Brosnan Forest.

### 2.2 Capture

During May 2013–August 2015, a total of 36 female white‐tailed deer (≥1 year old) were immobilized via a 2 cc transmitter dart (Pneu‐dart Inc., Williamsport, Pennsylvania, USA) containing a Xylazine (Lloyd Laboratories, Shenandoah, Iowa; 100 mg/ml given at a rate of 2.2 mg/kg) and Telazol (Fort Dodge Animal Health, Fort Dodge, Iowa; 100 mg/ml given at a rate of 4.5 mg/kg) mixture. Deer were fit with an ATS G2110D GPS Collar (Advanced Telemetry Systems, Isanti, Minnesota) tightened to within approximately two finger widths of the neck, allowing the collar to stay in the proper upright position, thereby improving data accuracy (D'Eon & Delparte, 2005). After processing was completed, a 3‐ml intramuscular injection of Tolazoline (Lloyd Laboratories, Shenandoah, Iowa; 100 mg/ml given at a rate of 6.6 mg/kg) was administered to act as a reversal to the Xylazine. All protocols involving animals were approved by the Auburn University Animal Care and Use Committee (PRN# 2013‐2205).

### 2.3 Data collection and manipulation

Collars were programmed to take GPS fixes at 30‐min intervals from 16 August to 1 December, recording position in UTM coordinates, date, time, altitude, fix status, satellites, position dilution of precision (PDOP), horizontal dilution of precision (HDOP), and temperature. Data were offloaded using ATS WinCollar software, and likely erroneous three‐dimensional fixes with PDOP >10 or HDOP >6 and two‐dimensional fixes with HDOP >3 were removed (D'Eon & Delparte, 2005; Lewis, Rachlow, Garton, & Vierling, 2007). Collared deer were eligible for hunter harvest beginning on 1 December each year, and harvested animals were taken to a central processing facility where harvest location and time, age, weight, and pregnancy status were recorded. All deer were aged via tooth wear and replacement (Severinghaus, 1949). Fetuses were removed from pregnant females, and date of conception was back calculated using a fetal aging scale (Hamilton, Tobin, & Moore, 1985).

All spatial data were classified using two separate approaches. First, following the classification system of previous studies (Beier & McCullough, 1990; D'Angelo et al., 2004; Holzenbein & Schwede, 1989; Kolodzinski et al., 2010; Labisky & Fritzen, 1998; Sawyer, 1981), we assigned each fix as being either prerut, rut, or postrut. Breeding periods on Brosnan Forest ranged from 16 August to 18 September, 19 September to 28 October, and 29 October to 1 December, respectively. These dates were determined such that the rut period would account for 80% of all conceptions previously documented on the study site (McCoy et al., 2013). Second, for all deer that conceived during our study, we created a covariate for days since conception for each fix ranging from 45 days prior to conception to 45 days postconception. We used 45 days as it would encompass movements up to and after conception and is approximately 2 weeks greater than the 28‐day estrous cycle of female white‐tailed deer (Plotka, Seal, Schmoller, Karns, & Keenlyne, 1977). Examining activity at both population breeding stages and relative to date of conception allowed us to compare movements of individuals to population‐level trends from this study and previously reported studies and to determine whether behaviors observed in this population were site specific or potentially generally representative of white‐tailed deer.

We evaluated six spatial metrics (movement rate, home range size, core area size, core area:home range ratio, probability of being outside the seasonal home range, and the probability of activity) that we hypothesized could be impacted by breeding‐related activity. Movement rate was calculated by finding the average Euclidean distance between consecutive half hour fixes throughout a single day (Labisky & Fritzen, 1998; Root, Fritzell, & Giessman, 1988; Webb, Gee, Strickland, Demarais, & DeYoung, 2010). Following Webb et al. (2010), only days with at least half of the possible consecutive fixes were utilized. We calculated home range and core use areas for each deer using the Brownian bridge method (Horne, Garton, Krone, & Lewis, 2007) for our study period (seasonal home range), as well as for the prerut, rut, and postrut periods. The 95% and 50% isopleths were used for the home range and core area estimates, respectively. Additionally, for females that conceived, home ranges and core area sizes were calculated for 13, 7‐day periods from 45 days before until 45 days after conception. The area of each home range and core area was calculated using ArcMap 10.2 (ArcMap version 10.2, ESRI Inc., Redlands CA, ESRI, 2013). Core area:home range ratio was calculated by dividing core area size by home range size and allowed for characterization of the intensity of an individual's use of space, where a ratio close to 1 indicated an individual was using its entire home range equally and a ratio close to 0 suggested that an individual was using small portions of its home range much more intensively (Monsarrat et al., 2013; Rotem, Berger, King, Bar, & Saltz, 2011). To find the probability of being outside the seasonal home range (SHR), each fix was classified as within or not within the SHR. The probability of a deer being outside the SHR provides information about an individual's propensity to travel outside of its home range and engage in exploratory movements (Kolodzinski et al., 2010). Probability of activity was calculated daily for each 30‐min interval within three separate periods: the 24‐hr day, diurnal hours, and nocturnal hours. Diurnal hours ranged from one half hour before sunrise until one half hour after sunset, with nocturnal being the remainder of the 24‐hr day. While white‐tailed deer are crepuscular by nature, their reproductive behaviors do not necessarily follow the same pattern. Therefore, crepuscular periods were not analyzed separately. Probability of activity was treated as a binary variable, and an individual was considered to be active in a 30‐min interval if the distance between consecutive locations was greater than the predetermined threshold distance of 51.78 m according to Sullivan, Ditchkoff, Collier, Ruth, and Raglin (2016).

Finally, movement paths of all individuals found to have conceived were inspected for excursions outside of the seasonal home range. Similar to previous studies (Karns, Lancia, DePerno, & Conner, 2011; Kolodzinski et al., 2010), an excursion was defined as (1) any series of fixes outside of the seasonal home range which extended >0.5 km, or (2) when half of an animal's daily fixes were outside of the seasonal home range. When an excursion was identified, the date and days from conception were recorded.

### 2.4 Data analysis

Movement rate, home range and core area size, and core area:home range ratio were analyzed relative to population breeding stages using linear regression (metric~rut stage), while likelihoods of activity and being outside the SHR were analyzed via logistic regression. Linear regression was used to evaluate variation in home range size, core area size, and core area:home range ratio relative to date of conception, while movement rate was analyzed via generalized additive modeling (metric~days to conception) with the model specified as a Gaussian distribution and a cubic regression spline smoother (Wood, 2006). Probability of activity and probability of being outside the SHR were also analyzed via generalized additive modeling but with distributions specified as binomial. We estimated the probability of an excursion occurring each day relative to conception via a general linear model with a second‐degree polynomial and used a generalized linear model to determine whether age class influenced the probability of an excursion. All analyses were conducted in R (v3.1.3; R Core Team, 2015).

## 3 Results

During the study, the 36 collars had an average fix success rate, after data censoring, of 87.61% (*n *=* *163,508), while the stationary collars had an average fix success rate of 99.87% (*n *=* *792). Thirty‐two collared deer with an average age of 3 years (*SE* = 0.2) were harvested by hunters following the study period, and conception was documented in 21 of these animals. Fetuses ranged from 40 to 114 days postconception with calculated conception dates from 11 September to 2 November. We also found that 80.95% of conceptions occurred during the rut period.

**Figure 1:**
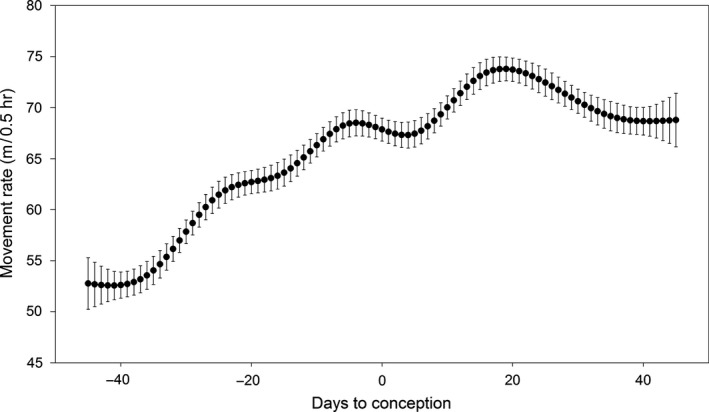
Movement rate of female white‐tailed deer at Brosnan Forest, SC relative to their date of conception, 2013–2015. Error bars represent *SE*

**Figure 2:**
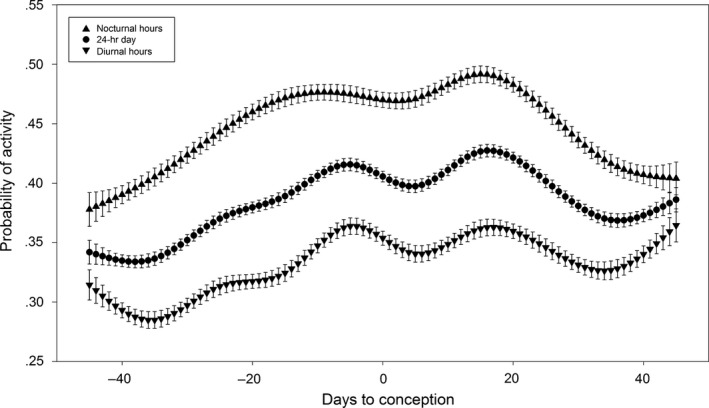
Probability of activity during any given 30‐min interval during the 24‐hr day, diurnal hours, and nocturnal hours relative to date of conception for female white‐tailed deer at Brosnan Forest, SC, 2013–2015. Error bars represent *SE*

**Figure 3:**
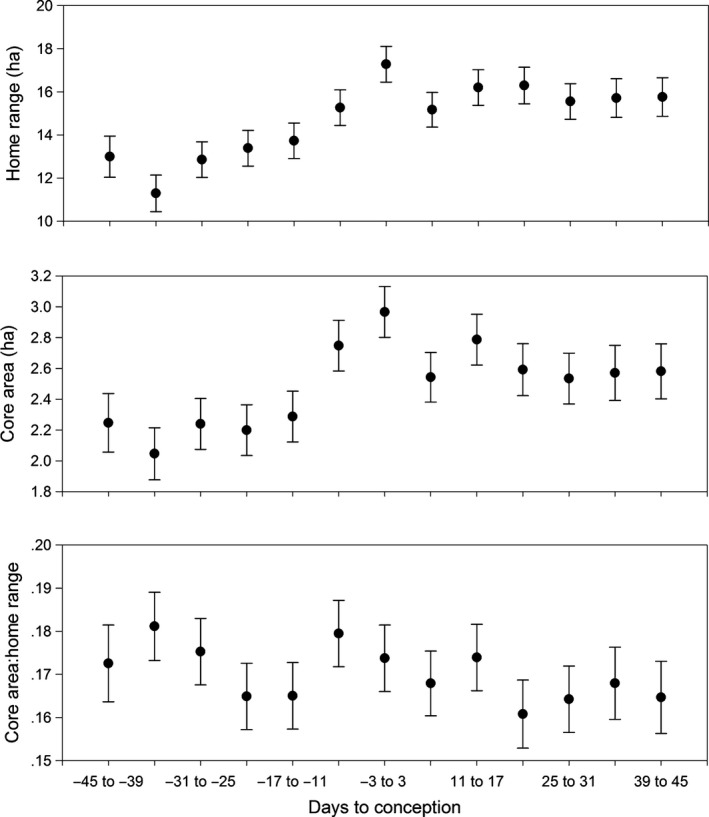
Home range, core area, and core area:home range ratio of female white‐tailed deer at Brosnan Forest, SC, every week relative to date of conception, 2013–2015. Error bars represent *SE*

There was a general trend of increasing activity from the prerut to the rut when examining movement rate, probability of activity, probability of being outside the SHR, home range size, core area size, and core area:home range (Table 1). Movement rate, probability of activity, and probability of being outside the SHR all increased from prerut to rut, and while movement rate and probability of activity declined from rut to postrut, probability of being outside the SHR continued to rise during this period. The space use metrics of core area and home range size both increased from prerut to rut, while core area:home range did not. While home range size continued to increase from rut to postrut, core area size and core area:home range values declined. When examined relative to date of conception, there was a tendency for activity metrics (movement rate and probability of activity) to steadily increase during the 40 days prior to conception, level off or slightly decrease in the days around conception, and then increase once again until 15–20 days following conception (Figures 1 and  2). Home range (17.284 ha, *SE* = 0.826) and core area (2.966 ha, *SE* = 0.165) were both greatest during the weeks surrounding conception, but the core area:home range surrounding conception was not found to be different from any other period (Figure 3).

**Table 1:** Mean measures of movement rate, probability of activity during any given 30‐min interval, probability of being outside of the seasonal home range, home range size, core area size, and core area: home range ratio for female white‐tailed deer at Brosnan Forest, SC during prerut, rut, and postrut periods of 2013–2015

	Prerut				Rut				Postrut			
Metric	*n*	$\overset{¯}{X}$	*SE*	Significance^a^	*n*	$\overset{¯}{X}$	*SE*	Significance^a^	*n*	$\overset{¯}{X}$	*SE*	Significance^a^
Movement rate (m/0.5 hr)	1,187	54.6	0.5	A	1,375	68.0	0.5	B	1,108	66.5	0.6	C
Probability of activity	48,869	0.327	0.002	A	56,932	0.419	0.002	B	47,102	0.368	0.002	C
Probability outside SHR	52,791	0.020	0.001	A	60,864	0.036	0.001	B	49,239	0.046	0.001	C
Home range (ha)	36	18.319	0.949	A	36	23.004	0.949	B	36	23.367	0.949	B
Core area (ha)	36	3.481	0.199	A	36	4.420	0.199	B	36	2.808	0.199	A
Core area:home range	36	0.193	0.006	A	36	0.193	0.006	A	36	0.163	0.006	B

a Different letters within a row indicate statistically significant differences between groups (*p* < .05).

**Figure 4:**
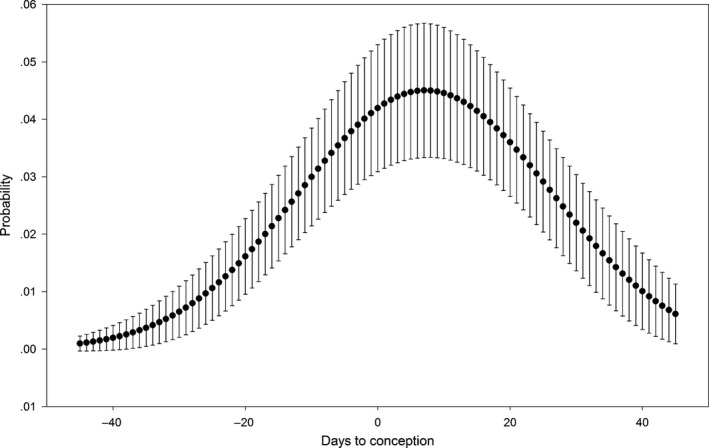
Probability of a female white‐tailed deer at Brosnan Forest, SC performing an excursion relative to date of conception, 2013–2015. Error bars represent *SE*

**Figure 5:**
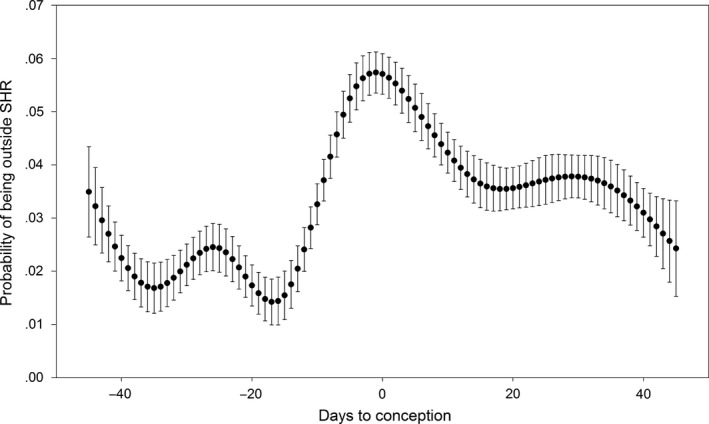
Probability of a female white‐tailed deer at Brosnan Forest, SC being outside its seasonal home range (SHR) relative to date of conception, 2013–2015. Error bars represent *SE*

We identified 23 excursions from the 21 deer that conceived with 2, 14, and 7 excursions performed during the prerut, rut, and postrut periods, respectively. Excursions ranged from 43 days before to 36 days postconception (Table 2), and the probability of a deer going on an excursion on a particular day increased prior to conception and peaked at 0.045 (*SE* = 0.012), 7 days postconception (Figure 4). We found no evidence that age impacted whether a deer performed an excursion (*p *=* *.125, *SE* = 0.378). The probability of being outside the SHR peaked surrounding conception, with the maximum observed value of 0.057 (*SE* = 0.004) occurring 1 day prior to conception (Figure 5).

**Table 2:** Deer age, date, days to conception, and breeding stage of the population for each excursion documented in adult female white‐tailed deer at Brosnan Forest, SC, 2013–2015

Deer	Age	Date	Days to conception^a^	Period
1	1.5	10/19	−4	rut
		10/26	3	rut
		11/9	17	postrut
		11/23	31	postrut
2	2.5	9/24	−3	rut
3	2.5	11/30	34	postrut
4	2.5	10/7	−1	rut
		10/8	0	rut
		10/17	9	rut
5	4.5	9/20	−43	rut
6	4.5	9/14	−23	prerut
		10/10	3	rut
		10/16	9	rut
		10/22	15	rut
		11/12	36	postrut
7	2.5	9/15	0	prerut
8	5.5	9/27	−2	rut
		10/9	10	rut
9	2.5	10/26	−3	rut
		11/5	7	postrut
		11/25	27	postrut
10	3.5	9/30	−1	rut
11	5.5	10/31	26	postrut

a Negative values refer to days prior to conception and positive values refer to days postconception.

## 4 Discussion

Our results suggest that adjustments in female behavior and space use appear to be closely linked to conception. When examining the spatial metrics relative to breeding stages, an increase in movement rate and probability of activity from prerut to rut suggests that females were actively advertising their presence during the breeding season (D'Angelo et al., 2004; Labisky & Fritzen, 1998; Sawyer, 1981). The conclusion that females are increasing movement to serve as a method of advertisement is supported by an increase in both metrics from approximately 40 days before until just prior to conception. Increases in both home range and core area from prerut to rut, paired with no change in the core area:home range ratio over the same time period, suggest that deer were expanding movements over a larger area while using this area uniformly (Monsarrat et al., 2013; Rotem et al., 2011). Our results indicate that peak home range and core area sizes were observed near conception, suggesting that the increase was driven by breeding behaviors, and the peak during the rut period occurs due to most deer breeding within that window of time. Such space use is directly opposed to the behavior expected in a sit‐and‐wait strategy where deer would concentrate movement into a portion of the core area (Beier & McCullough, 1990; Holzenbein & Schwede, 1989; Ivey & Causey, 1981).

Perhaps the trends that most clearly demonstrate the connection between behavioral changes and breeding are those seen through the probability of being outside the seasonal home range and the daily probability of performing an excursion. The probability of being outside the seasonal home range increased approaching conception and declined postconception, suggesting that deer were attempting to advertise their presence to as many potential mates as possible (Kolodzinski et al., 2010). Additionally, while the peak probability of an excursion was after conception, the cluster of excursions within 3 days of conception suggests that excursive behaviors and increases in activity before conception were related to reproductive activities. However, the occurrence of several excursions both before and after conception indicates that breeding is likely not the only driver of excursions, even when occurring during the breeding season. An additional potential explanation for the observed behaviors is harassment by rutting males or isolation during the tending bond (Kilpatrick & Lima, 1999; Kolodzinski et al., 2010). However, we find this unlikely as several excursions were very short in duration, lasting only a few hours, whereas the tending bond has been reported as lasting 24–72 hr (DeYoung & Miller, 2011). Furthermore, while pairs isolate themselves during the tending period, we believe it is unlikely that the doe would forfeit the safety of her home range for this behavior. Unfortunately, we do not have data that would allow us to definitively determine whether observed excursions occurred prior to, or as a result of, contact with a male. Future studies would require proximity loggers to answer such questions. The potential also exists that these behaviors could be a response to hunting pressure as opposed to reproductive activities (Kilpatrick & Lima, 1999; Kolodzinski et al., 2010). Hunting pressure on our study site was very consistent both spatially (a lack of large refuge area) and temporally (hunting occurred equally throughout the week and season) due to the properties role as an outdoor recreational facility for large conference groups (Sullivan, 2016). Therefore, we deem it unlikely that these temporally specific behaviors were a response to the long and sustained hunting seasons that took place on this property during our research.

Increases in movement rate and probability of activity postconception were unexpected, as it is not advantageous to maintain mate‐seeking behavior once breeding has occurred (Labisky & Fritzen, 1998). However, it is possible that a female is unaware whether she has conceived until approximately 20 days postconception and therefore behaves as though she will be entering a second estrus. In ruminants, pregnancy may be recognized following the release of interferon tau, which signals the stoppage of luteolysis, and subsequent increase in progesterone levels (Bazer, Ott, & Spencer, 1998; Demmers, Kaluz, Deakin, Jabbour, & Flint, 1999; Spencer, Burghardt, Johnson, & Bazer, 2004). It is likely these changes in progesterone level, referred to as the pregnancy hormone (Spencer et al., 2004), result in behavioral changes for female white‐tailed deer. Plotka et al. (1977) observed a continuous increase in progesterone levels of bred female white‐tailed deer from the onset of estrus through 40 days postestrus, with a significant increase from 10 to 20 days postestrus. However, had a deer not conceived, progesterone levels would have been expected to decline beginning approximately 14 days postestrus (Plotka et al., 1977), a trend observed throughout Cervidae (Chapple, English, & Mulley, 1993; Kelly, McNatty, & Moore, 1985; Liu, Cheng, Huang, & Yu, 2002). We hypothesized that the maintenance of elevated levels of progesterone from days 14 to 40 postconception enables a female to identify her pregnancy and cease breeding behaviors, yet manipulative experiments would be required to confirm this link.

Due to the moderate deer density and balanced male:female ratio at Brosnan Forest, previous studies would suggest that the sit‐and‐wait strategy would be the most efficient means of breeding for female white‐tailed deer (Holzenbein & Schwede, 1989; Labisky & Fritzen, 1998). However, behavioral patterns observed during the three breeding stages closely resembled those reported by Labisky and Fritzen (1998) for a low‐density deer population (five deer/km^2^). As females should have experienced no difficulty finding a mate on our study site, we suggest that the conclusion of Labisky and Fritzen (1998) that excursions serve only as a last resort to find a mate may not be generally applicable. Similar to Kolodzinski et al. (2010), we conclude that excursive behavior in female white‐tailed deer is an adaptive behavior to maximize the quality of the pool of potential mates and is an expression of female mate choice.

While it appears that female white‐tailed deer are performing excursive behavior as a form of mate choice, it is not likely that these excursions are an attempt to locate a particular mate. Stopher et al. (2011) rejected the notion of females using excursions to reach preferred males in red deer as the excursions appeared random. Furthermore, as males are highly mobile during the rut, the likelihood of an excursion allowing a female to locate a specific male is low (DeYoung & Miller, 2011; Labisky & Fritzen, 1998). Instead, a straight line search, which has been observed for excursions, would maximize the likelihood of encountering any given male, while an intensive search would be required to locate a particular male (Zollner & Lima, 1999). The impracticality of a female utilizing an excursion to locate a specific male suggests that excursive behaviors may be an attempt to incite competition by alerting more males of the female's presence, as suggested by Cox and Le Boeuf (1977).

Our results provide insight into the movement ecology and behavioral strategies employed by reproductively active female white‐tailed deer, and our examination of spatial metrics relative to an individual's date of conception provides a more detailed understanding of breeding behaviors. While the results of the breeding stage analysis in this study supported trends seen relative to date of conception, they failed to identify unique behaviors which provide a greater degree of insight into possible implications of animal activities. Therefore, we recommend that future behavioral studies focus on events at the individual scale as opposed to population‐level analyses.

## Conflict of Interest

None declared.
